# Discordant nodal staging identifies intermediate-risk group for overall survival in patients with cT3 oesophageal adenocarcinoma

**DOI:** 10.1007/s00330-019-06642-6

**Published:** 2020-02-13

**Authors:** Charles Carder, Patrick Fielding, Ashley Roberts, Kieran Foley

**Affiliations:** 1 Department of Clinical Radiology, University Hospital of Wales, Cardiff, UK; 2 Wales Research and Diagnostic Positron Emission Tomography Imaging Centre (PETIC), Cardiff University, Cardiff, UK; 3 Department of Clinical Radiology, Velindre University NHS Trust, Cardiff, UK; 4 Division of Cancer & Genetics, School of Medicine, Cardiff University, Cardiff, UK

**Keywords:** Oesophageal neoplasm, Lymph node, Neoplasm staging, Positron emission tomography, Endosonography

## Abstract

**Objectives:**

Oesophageal adenocarcinoma has a poor prognosis and relies on multi-modality assessment for accurate nodal staging. The aim of the study was to determine the prognostic significance of nodal concordance between PET/CT and EUS in oesophageal adenocarcinoma.

**Methods:**

Consecutive patients with oesophageal adenocarcinoma staged between 2010 and 2016 were included. Groups comprising concordant node–negative (C−ve), discordant (DC), and concordant node–positive (C+ve) patients were analysed. Survival analysis using log-rank tests and Cox proportional hazards model was performed. The primary outcome was overall survival. A *p* value < 0.05 was considered statistically significant.

**Results:**

In total, 310 patients (median age = 66.0; interquartile range 59.5–72.5, males = 264) were included. The median overall survival was 23.0 months (95% confidence intervals (CI) 18.73–27.29). There was a significant difference in overall survival between concordance groups (*X*^2^ = 44.91, df = 2, *p* < 0.001). The hazard ratios for overall survival of DC and C+ve patients compared with those of C−ve patients with cT3 tumours were 1.21 (95% CI 0.81–1.79) and 1.79 (95% CI 1.23–2.61), respectively. On multivariable analysis, nodal concordance was significantly and independently associated with overall survival (HR 1.44, 95% CI 1.12–1.83, *p* = 0.004) and performed better than age at diagnosis (HR 1.02, 95% CI 1.003–1.034, *p* = 0.016) and current cN-staging methods (HR 1.20, 95% CI 0.978–1.48, *p* = 0.080).

**Conclusions:**

Patients with discordant nodal staging on PET/CT and EUS represent an intermediate-risk group for overall survival. This finding was consistent in patients with cT3 tumours. These findings will assist optimum treatment decisions based upon perceived prognosis for each patient.

**Key Points:**

*• Clinicians are commonly faced with results of discordant nodal staging in oesophageal adenocarcinoma.*

*• There is a significant difference in overall survival between patients with negative, discordant, and positive lymph node staging.*

*• Patients with discordant lymph node staging between imaging modalities represent an intermediate-risk group for overall survival.*

**Electronic supplementary material:**

The online version of this article (10.1007/s00330-019-06642-6) contains supplementary material, which is available to authorized users.

## Introduction

Oesophageal cancer is newly diagnosed in over 9000 people in the United Kingdom (UK) each year and predominately of adenocarcinoma cell type, with around 7 in 10 diagnosed at an advanced stage [1]. Radiological staging is central to management, planning, and prognosis, and usually involves a combination of computed tomography (CT), positron emission tomography combined with CT (PET/CT), and endoscopic ultrasound (EUS) [2].

Lymph node metastases are a major prognostic indicator in oesophageal cancer [3, 4]. Nodal assessment therefore is a key factor in radiological staging but the accuracy of these individual modalities is suboptimal [5, 6]. EUS is generally regarded as the gold standard for assessment of regional lymph nodes, but controversy regarding the role of EUS in staging exists. Studies have shown limited benefits versus risk [7] whilst others suggest EUS can impact treatment decisions in 29% of patients [8] and reduce edge of radiotherapy field relapses when EUS measurements are used to define gross tumour volume [9].

There is a lack of studies investigating the prognostic significance of nodal concordance between imaging modalities in oesophageal cancer staging. Diagnostic confidence is improved when a node has malignant characteristics on more than one imaging modality. The high specificity and positive predictive value compared with low sensitivity and negative predictive value of CT and PET/CT [10, 11] mean that a lymph node is often considered to be involved if malignant characteristics are demonstrated on multiple modalities.

However, clinicians face a difficult diagnostic conundrum in cases where there are discordant findings of malignancy in lymph nodes between different modalities. Important treatment decisions often hinge on the classification of nodal metastases on PET/CT and EUS examinations. In a previous study, Dhupar et al [12] reviewed 615 patients with oesophageal cancer from a single centre who underwent oesophagectomy for survival outcomes based on concordance of staging investigations for nodal disease. In summary, the study found that patients with discordance in nodal staging between imaging modalities had a better overall survival than patients with positively concordant nodal staging. However, more data is required to validate these findings.

Therefore, the aim of this study was to determine the prognostic significance of nodal concordance between PET/CT and EUS in oesophageal adenocarcinoma.

## Materials and methods

Institutional review board approval was obtained for this study (reference 13//DMD5769). We performed a retrospective review of a prospectively collected database of patients with oesophageal adenocarcinoma in a regional upper GI cancer network comprising four health boards and eight different centres.

Consecutive patients (*n* = 420) were considered for this study. Inclusion criteria were patients with biopsy-confirmed adenocarcinoma of the oesophagus or gastro-oesophageal junction (GOJ) who were staged with contrast-enhanced CT, PET/CT, and EUS between 2010 and 2016. Exclusion criteria were a histological cell type other than adenocarcinoma, those who did not have PET/CT (*n* = 2) or EUS (*n* = 89; *n* = 71 because of M1 disease on PET/CT and *n* = 18 due to non-traversable stenotic tumour), patients with missing TNM staging data (*n* = 17), and patients with missing survival data (*n* = 2). Following exclusion criteria application, 310 patients were included. During the study period, patients were staged according to TNM 7th edition [13]. Use of the TNM 8th edition [14] would not have altered stage groupings.

### Staging pathway

Patients are usually diagnosed with oesophageal cancer following upper GI endoscopy and biopsy. Patients then undergo a contrast-enhanced CT of the thorax, abdomen, and pelvis to assess for distant metastases. If the patient was considered to have potentially curable disease after staging CT (i.e. absence of M1 disease), then more detailed staging with PET/CT and EUS was performed. EUS was not completed in patients with a non-traversable stenotic tumour and not performed in patients with confirmed M1 disease on PET/CT. The PET/CT and EUS protocols are available in the supplementary material.

### Clinical T-stage

Following multi-disciplinary team (MDT) review, clinical T-stage (cT-stage) was assigned to each patient following consideration of the contrast-enhanced CT and EUS findings, with the latter being considered the most accurate modality for cT-stage [15].

### Definition of positive lymph node metastasis

On PET/CT, nodes were classed as involved if identified on the CT component and showed FDG uptake appreciably higher than background values. No specific standardised uptake value was used to diagnose regional nodes because this decision is subjective and multi-factorial. Lymph nodes considered physiological or related to an alternative aetiology were excluded from the N-stage. On EUS, the criteria for malignant lymphadenopathy specified a hypoechoic pattern, spherical contour, distinct border, and a short-axis diameter of 6 mm or more. PET/CT and EUS cN-staging were collected from the clinical radiology reports which were used to decide the subsequent treatment. Overall clinical N-stage (cN-stage) was assigned following MDT review after consideration of the combined CT, PET/CT, and EUS findings.

### Definition of concordance

**Fig. 1:**
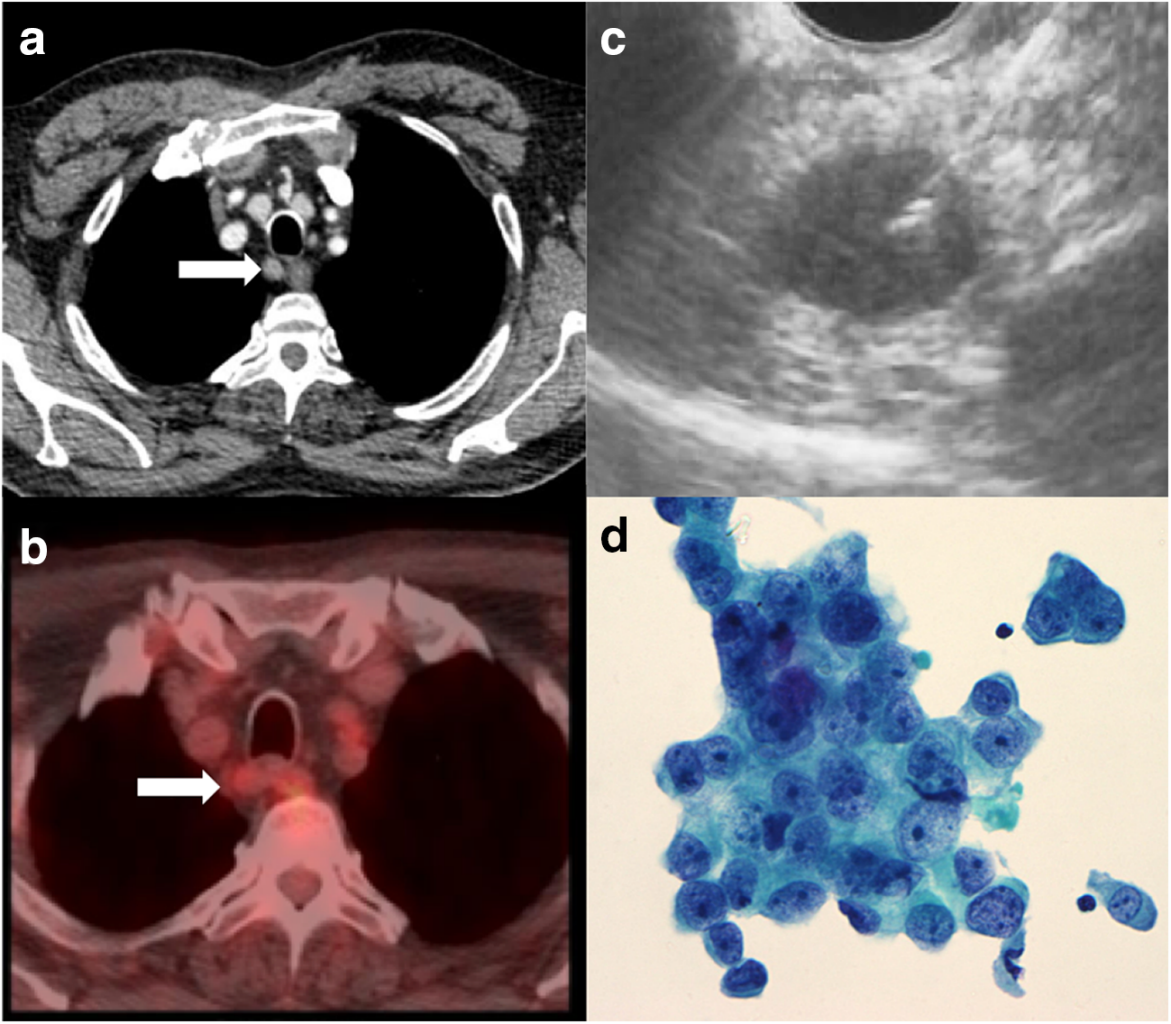
Clinical example of discordant radiological lymph node staging. A patient with a primary distal oesophageal adenocarcinoma had a 9-mm lymph node (white arrows) on (**a)** a contrast-enhanced CT and (**b)** a PET/CT with minimally increased SUV (2.6) compared with background. An (**c)** EUS was performed which shows a malignant appearance (8 mm, round and hypoechoic), and a fine needle aspiration (FNA) was performed. The (**d)** FNA with papanicolaou stain at × 40 magnification showed adenocarcinoma cells from the lymph node (large pleomorphic cells with prominent nucleoli in a cohesive group)

Three classifications were defined for this study. Nodal concordance was defined as either negative (C−ve) or positive (C+ve). C−ve patients had cN0 disease on both PET/CT and EUS and C+ve patients had cN+ disease on both modalities. Discordant patients (DC) had cN0 disease on one modality but not the other (Fig. 1). Further subgroups were constructed based on modality findings; PET/CT−ve:EUS+ve and EUS−ve:PET/CT+ve.

### Survival data

The primary outcome of the study was overall survival, defined as the length of survival following diagnosis until death or date of last follow-up. Survival data was obtained from the Cancer Network Information System database. Patients are followed up at regular intervals following radical treatment, every 3 months for the first year and then 6-monthly thereafter for the next 4 years.

### Statistical analysis

Descriptive statistics were expressed as frequency (percentage) for categorical variables and median (interquartile range (IQR)) for continuous variables. Differences between categorical variables and continuous variables were assessed using chi-square tests and Mann-Whitney *U* tests, respectively. Median overall survival was estimated using the Kaplan-Meier life-table method [16]. Mean overall survival was calculated when median overall survival was not reached. Cumulative survival curves were generated and differences between groups evaluated with the log-rank test. The prognostic significance of cT-stage and cN-stage were evaluated using this method. Hazard ratios were calculated with a Cox proportional hazards model and compared with the baseline group. A subgroup analysis for curative versus palliative treatment groups was pre-specified and performed separately. Multivariable analysis evaluated whether age at diagnosis, nodal concordance, or current cN-staging methods were the better predictor of overall survival. Statistical analysis was performed using SPSS v23.0 (IBM). A *p* value < 0.05 was considered statistically significant.

## Results

The baseline characteristics of included patients are detailed in Table 1. The median age of the cohort was 66.0 years (IQR 59.5–72.5). The median overall survival was 23.0 months (95% confidence intervals (CI) 18.73–27.29). One-, 2-, and 5-year overall survival rates were 73.5% (*n* = 228/310; 95% CI 73.45–73.55), 48.4% (*n* = 150/310; 95% CI 48.35–48.45), and 13.5% (*n* = 42/310; 95% CI 13.44–13.55), respectively. The median follow-up was 63.0 months (95% CI 57.97–68.04).

**Table 1:** Baseline characteristics of patient cohort

Clinical variable	Frequency (%)
Gender	
Male	264 (85.2)
Female	46 (14.8)
Tumour location	
Oesophagus	158 (51.0)
Mid oesophagus	27 (17.1)
Distal oesophagus	131 (82.9)
GOJ	152 (49.0)
Siewert type I	60 (39.5)
Siewert type II	44 (28.9)
Siewert type III	48 (31.6)
Grade of differentiation	
Well	21 (6.8)
Moderate	97 (31.3)
Poor	116 (37.4)
GX	76 (24.5)
cT-stage	
T1	29 (9.4)
T2	34 (11.0)
T3	202 (65.2)
T4a	41 (13.2)
T4b	4 (1.3)
cN-stage	
N0	132 (42.6)
N1	96 (31.0)
N2	54 (17.4)
N3	28 (9.0)
cM-stage	
M0	310 (100)
Treatment	
Radical	222 (71.6)
NACT	94 (42.3)
dCRT	56 (25.2)
Surgery alone	48 (21.6)
NACRT	20 (9.0)
EMR	4 (1.8)
Palliative	88 (28.4)

*GOJ*, gastro-oesophageal junction; *GX*, grade not assessed; *cT-stage*, clinical tumour stage; *cN-stage*, clinical regional nodal stage; *cM-stage*, clinical metastatic stage; *NACT*, neoadjuvant chemotherapy; *dCRT*, definitive chemoradiotherapy; *NACRT*, neoadjuvant chemoradiotherapy; *EMR*, endoscopic submucosal resection

A large number of patients (*n* = 89) were initially excluded because of missing EUS data; therefore, comparison of baseline characteristics was performed in these patients. Age (*t* = 1.518, mean difference 1.738 (95% CI − 0.513–3.988), *p* = 0.130), gender (*X*^2^ 0.003, df 1, *p* = 0.957), and tumour location (*X*^2^ 0.239, df 1, *p* = 0.625) were not significantly different between patient groups with and without EUS staging.

### Prognostic significance of nodal concordance between PET/CT and EUS

**Fig. 2:**
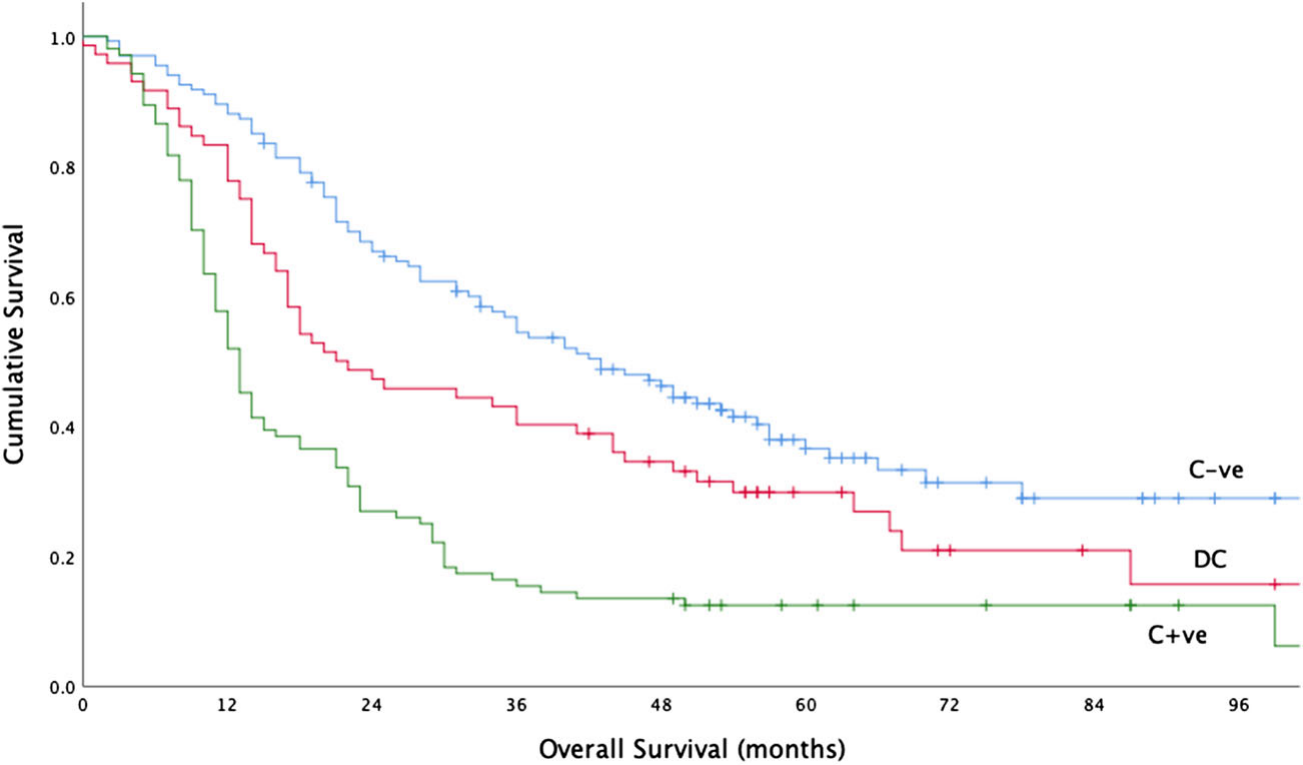
KM plot showing cumulative survival curves for C−ve, DC, and C+ve patients (*X*^2^ 44.91, df 2, *p* < 0.001)

The median overall survival for C−ve, DC, and C+ve patients were 43.0 months (95% CI 33.22–52.78), 21.0 months (95% CI 9.36–32.64), and 13.0 months (95% CI 11.25–14.76), respectively. There was a significant difference in overall survival between groups (*X*^2^ 44.91, df 2, *p* < 0.001) (Fig. 2). Furthermore, there were significant differences between C−ve and DC groups (*X*^2^ 5.18, df 1, *p* = 0.023) and DC and C+ve groups (*X*^2^ 11.11, df 1, *p* = 0.001). The hazard ratios for overall survival of DC and C+ve patients compared with those of C−ve patients overall were 1.46 (95% CI 1.04–2.06) and 2.66 (95% CI 1.97–3.60), respectively. This suggests that patients with discordant nodal staging between PET/CT and EUS represent an intermediate-risk group for overall survival.

### Prognostic significance of cT-stage and cN-stage

In this cohort, cT-stage (*X*^2^ 54.12, df 4, *p* < 0.001) and cN-stage (*X*^2^ 48.85, df 3, *p* < 0.001) were significantly associated with overall survival. The hazard ratios for overall survival of N1, N2, and N3 stages compared with those of N0 were 1.57 (95% CI 1.14–2.15), 3.13 (2.19–4.49), and 2.60 (1.65–4.09), respectively. Survival statistics are presented for each cT-stage in Table 2.

**Table 2:** Comparison of cT-stage between negative concordance, discordance, and positive concordance groups

T-stage	Concordance	Frequency (%)	Median OS (months) (95% CI)	*X*^2^	df	*p* value
T1 (*n* = 29)	C−ve	29 (100)	*79.22 (64.85–93.59)	-	-	-
	DC	0	-			
	C+ve	0	-			
T2 (*n* = 34)	C−ve	25 (73.5)	40.0 (25.31–54.69)	2.44	2	0.295
	DC	5 (14.7)	67.0 (67.0–67.0)			
	C+ve	4 (11.8)	9.0 (7.04–10.96)			
T3 (*n* = 202)	C−ve	71 (35.1)	33.0 (24.74–41.26)	10.19	2	0.006
	DC	61 (30.2)	22.0 (7.79–36.21)			
	C+ve	70 (34.7)	14.0 (7.85–20.15)			
T4a (*n* = 41)	C−ve	8 (19.5)	16.0 (6.30–25.70)	4.29	2	0.117
	DC	5 (12.2)	15.0 (8.56–21.44)			
	C+ve	28 (68.3)	12.0 (10.52–13.48)			
T4b (*n* = 4)	C−ve	1 (25.0)	*41.0 (41.00–41.00)	3.34	2	0.188
	DC	1 (25.0)	*8.0 (8.00–8.00)			
	C+ve	2 (50.0)	*6.5 (5.52–7.48)			

*Mean OS; *OS*, overall survival; *X*^*2*^, chi-square statistic; *df*, degrees of freedom; *C−ve*, negative node concordance; *DC*, nodal discordance; *C+ve*, positive node concordance

**Fig. 3:**
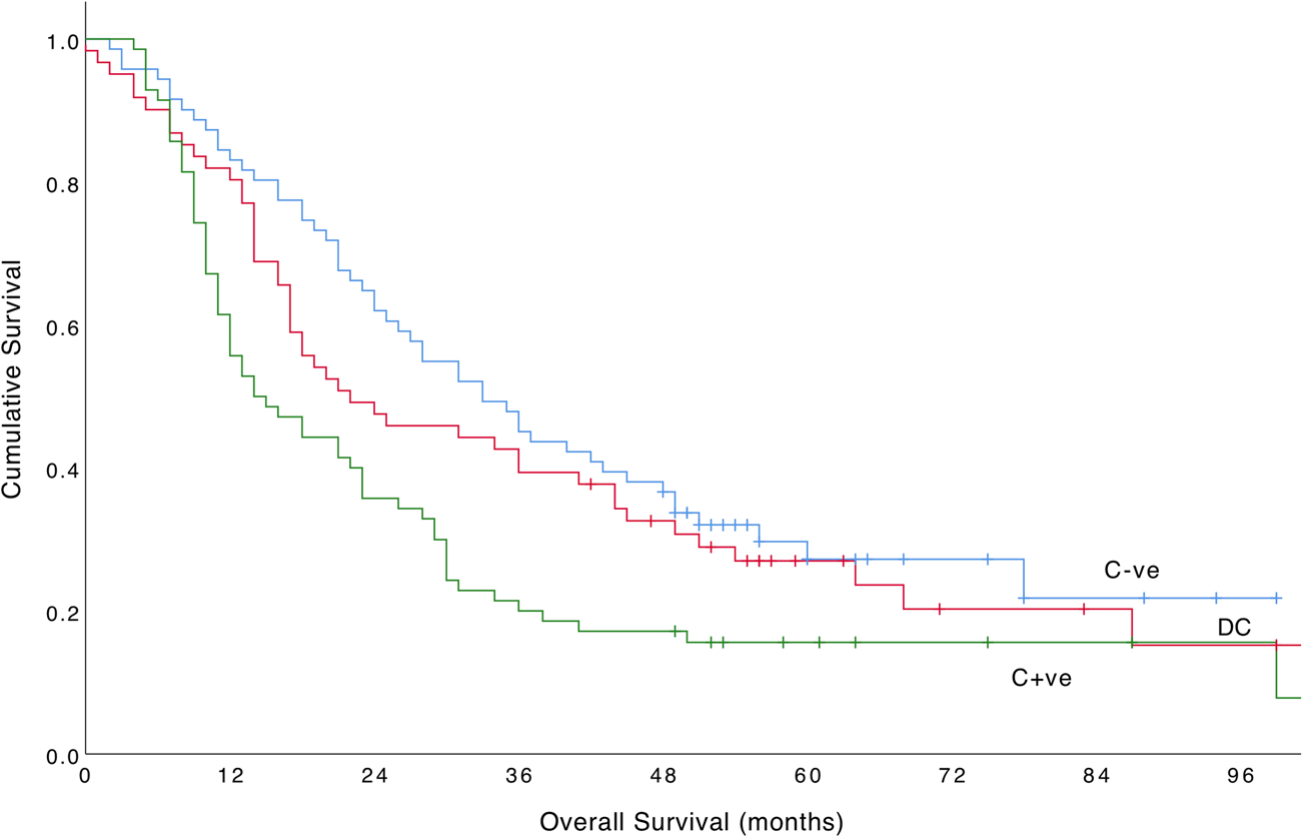
Cumulative survival curves for C−ve, DC, and C+ve patients with cT3 tumours (*X*^2^ 10.19, df 2, *p* = 0.006)

There was a significant difference between C−ve, DC, and C+ve patients staged with cT3 tumours (*X*^2^ 10.19, df 2, *p* = 0.006) (Fig. 3). cT3 was significantly associated with overall survival but other cT-stages were not, likely due to the small numbers included. The hazard ratios for overall survival of DC and C+ve patients compared with those of C−ve patients with cT3 tumours were 1.21 (95% CI 0.81–1.79) and 1.79 (95% CI 1.23–2.61), respectively. No significant difference in overall survival was found between C−ve and DC (*X*^2^ 0.94, df 1, *p* = 0.33), groups but there was a significant difference between DC and C+ve (*X*^2^ 3.19, df 1, *p* = 0.048) groups, suggesting that DC patients with cT3 tumours have similar survival to C−ve patients.

### Differences between PET/CT and EUS nodal staging

**Fig. 4:**
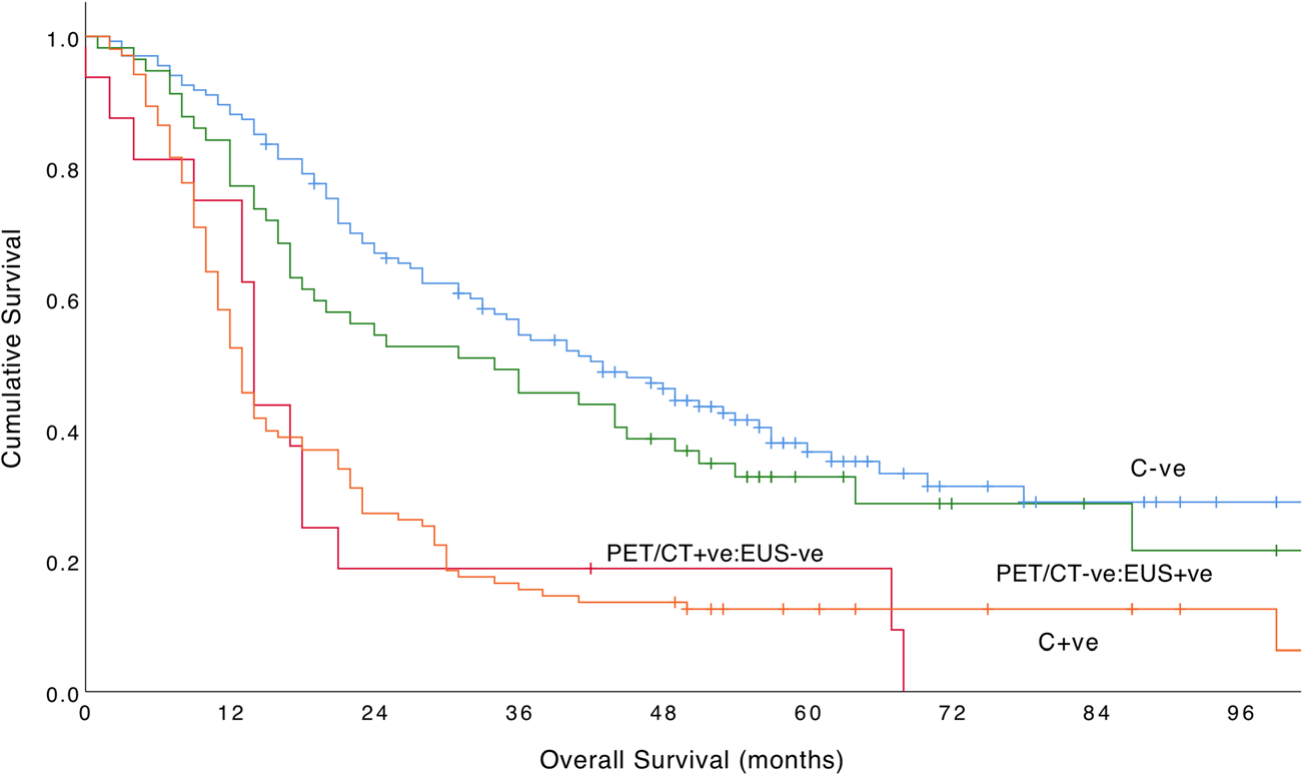
Cumulative survival curves for C−ve, PET/CT−ve:EUS+ve, EUS-ve:PET/CT+ve, and C+ve patients (*X*^2^ 50.99, df 3, *p* < 0.001)

The relationship between PET/CT and EUS N-staging was examined by creating two groups: (1) PET/CT−ve:EUS+ve and (2) EUS-ve:PET/CT+ve. In total, 16/310 patients (5.2%) had PET/CT cN+ and EUS cN0 disease. Conversely, 57/310 patients (18.4%) had EUS cN+ and PET/CT cN0 disease. There was a significant difference in frequency between these two groups (*X*^2^ 97.29, df 1, *p* < 0.001). In addition, there were significant differences between C−ve, PET/CT−ve:EUS+ve, EUS-ve:PET/CT+ve, and C+ve groups (*X*^2^ 50.99, df 3, *p* < 0.001) (Fig. 4). The median overall survival for the EUS-ve:PET/CT+ve group was 14.0 months (95% CI 12.70–15.30) and 34.0 months (95% CI 13.92–54.08) for the PET/CT−ve:EUS+ve group. There was a significant difference between PET/CT−ve:EUS+ve and EUS-ve:PET/CT+ve groups (*X*^2^ 6.50, df 1, *p* = 0.011) suggesting patients with PET/CT−ve:EUS+ve nodes had a better outcome, although the numbers in the PET/CT+ve:EUS-ve group were relatively low.

### Subgroup analysis

With different treatment regimens included in this study, a subgroup analysis compared the prognostic significance of nodal concordance between patients treated with radical versus palliative treatments. There was a significant difference in overall survival between C−ve, DC, and C+ve nodal concordance when adjusting for treatment intent (*X*^2^ 13.88, df 1, *p* = 0.001), demonstrating that the discordant group remained at intermediate risk in both radical and palliative settings. The median overall survival for patients treated with radical versus palliative intent was 37.0 months (95% CI 28.77–45.23) and 12.0 months (95% CI 10.04–13.97), respectively. Furthermore, there were no significant differences in overall survival (*X*^2^ 6.977, df 4, *p* = 0.137) between radical treatment groups (neoadjuvant chemotherapy (NACT), definitive chemoradiotherapy (dCRT), surgery alone, neoadjuvant chemoradiotherapy (NACRT), and endoscopic mucosal resection (EMR)).

The hazard ratios for overall survival of DC and C+ve patients compared with those of C−ve in patients treated with radical intent were 1.24 (95% CI 0.84–1.84) and 1.75 (95% CI 1.16–2.64), respectively. In palliative patients, the hazard ratios for overall survival of DC and C+ve patients compared with those of C−ve patients treated palliatively were 3.13 (95% CI 1.46–6.67) and 2.38 (95% CI 1.28–4.42), respectively. These results show that the overall survival of DC patients more closely matched C−ve patients when treated with radical intent, but the opposite effect was true when treated palliatively.

### Multivariable analysis

On multivariable analysis, age at diagnosis, nodal concordance, and cN-stage were entered into a multi-variable model. Nodal concordance was significantly and independently associated with overall survival (HR 1.44, 95% CI 1.12–1.83, *p* = 0.004) and performed better than age at diagnosis (HR 1.02, 95% CI 1.003–1.034, *p* = 0.016) and current cN-staging methods (HR 1.20, 95% CI 0.978–1.48, *p* = 0.080).

## Discussion

This study has shown that patients with discordant nodal staging on PET/CT and EUS represent an intermediate-risk group for overall survival. This finding is important when deciding upon the optimum treatment decision based upon the perceived risk stratification for each patient [17].

Clinicians are commonly faced with results of discordant lymph node staging between imaging modalities. Accurate detection of lymph node metastases is important for staging and prognosis, and their detection changes treatment decisions. In this cohort, we have demonstrated the prognostic significance of cT- and cN-stage. However, clinical ambiguity is introduced into decision pathways if two modalities (PET/CT and EUS) are discordant. This ambiguity can create diagnostic and management uncertainty for clinicians who must judge the most appropriate treatment considering the best interests of each patient. In such cases, a discordant lymph node is often considered likely to be metastatic given the incidence of metastases with advanced cT-stage [3] and the higher specificity and positive predictive value of CT and PET [11].

These results are consistent with data from a cohort of oesophageal cancer patients who underwent oesophagectomy [12]. This study also described an intermediate-risk group comprising patients with discordant staging investigations, in which DC patients had better over survival than C+ve patients. Despite the findings of our current study, the literature surrounding the prognostic significance of discordant lymph node staging is sparse.

Importantly, this study has further shown the significant overall survival differences between C−ve, DC, and C+ve groups in patients with cT3 tumours. These data could be used to support clinical decisions in patients with cT3 tumours, which is the most common stage of oesophageal tumours at presentation [18]. Overall survival for patients with cT4 tumours was not found to be statistically significant between cT-stage groups, but this is likely to be an effect of small patient numbers in this subgroup. Patients with nodal discordance had an overall survival probability more closely aligned to patients with C−ve nodes, suggesting that in these cases, clinicians should place the findings in a wider clinical context and perhaps consider more radical treatment.

The subgroup analysis found that discordant nodal staging had a better prognosis in patients treated with radical intent but a worse prognosis in patients treated palliatively. These findings are likely to be biased by the retrospective nature of this study and influenced by the effects of treatment, which are broadly based on a number of clinical, physiological, radiological, and pathological factors. Endoscopic mucosal resection (EMR) is available for patients with limited early-stage disease. No survival benefit from EMR was demonstrated in this study, but only four EMR patients were included in this cohort. Fit, young patients may have been treated more aggressively than those with a significant number of comorbidities. In the latter cases, clinicians may have decided that the discordance added further uncertainty that the effects of radical treatment would be beneficial. This confounding bias cannot be adjusted for in retrospective studies, and these findings will need to be validated in prospective studies.

The number of patients classified with positive lymph nodes (cN+) is often higher on EUS than on PET/CT [15], and this trend was shown again here. PET imaging is limited in its ability to detect small and peri-tumoural lymph node metastases because of the relatively large spatial resolution and reliance on co-registration for anatomical definition [19]. In addition, the positive correlation between primary tumour and lymph node uptake means that metastases are less likely to be detected if the primary tumour demonstrates low FDG uptake. Overall, recent data has shown that the sensitivity of CT, PET/CT, and EUS is poor (39.7%, 35.3%, and 42.6%, respectively) [5], which is consistent with data from other specialist oesophageal cancer centres [6] and different thoracic tumour sites such as non–small cell lung cancer [20].

Similarly, there was a significant difference in overall survival between PET/CT−ve:EUS+ve and EUS-ve:PET/CT+ve groups, suggesting that patients with PET/CT−ve:EUS+ve discordant nodes had a better outcome, although the numbers in the PET/CT−ve:EUS+ve were low. Overall, these results show that clinicians should refrain from instinctively concluding that a discordant lymph node is metastatic. False-positive rates of CT, PET/CT, and EUS range between 15 and 30% for cN-staging [10].

New methods to improve lymph node metastasis staging accuracy are required because radiological techniques alone are unlikely to improve sufficiently in the near future. A high proportion of micro-metastases (82%) have been detected in normal-sized lymph nodes of patients with oesophageal cancer [5], and it is believed that micro-metastases are associated with a poorer clinical outcome [21], although the evidence is conflicting. At present, micro-metastases cannot be visualised on current imaging modalities.

The false-positive rates of each modality and high proportion of metastases highlight that lymph node staging should be considered within a multi-disciplinary context, with factors such as lymph node location and number, grade of primary tumour differentiation, and underlying tumour biology known to have prognostic significance [22].

### Strengths

This study includes a large consecutive cohort of fully staged patients from a large regional upper GI cancer network serving a population of approximately 1.5 million. The overall survival data are robust and no patient was lost to follow-up. A comparison of cT-stage and radical versus palliative care treatment subgroups demonstrated that the prognostic significance of discordant lymph nodes was consistent.

### Limitations

As discussed above, retrospective studies are likely to be hindered by confounding biases. Individual lymph nodes were not examined; rather, the cN-stage between modalities was compared on a per-patient basis. Histopathological correlation was not possible in the majority of the cohort because relatively few patients are suitable for surgical resection. Contrast-enhanced CT data were not analysed in this study because it is known that the accuracy of CT N-staging is poor and the PET/CT has a CT component which was used at the time of reporting to evaluate individual lymph nodes. Lastly, squamous cell carcinoma was not included because this study focussed on adenocarcinoma, the most prevalent histological cell type in European and North American countries [23].

In conclusion, this study has shown that patients with discordant nodal staging on PET/CT and EUS represent an intermediate-risk group for overall survival and have better survival rates than those with positively concordant lymph nodes. This finding was consistent in patients with cT3 tumours and is important when deciding upon the optimum treatment based upon the perceived prognosis for each patient. These results should be validated in prospective studies.

## Electronic supplementary material

ESM 1 (DOCX 24 kb)

## Abbreviations

C−ve: Concordant negative nodal status
C+ve: Concordant positive nodal status
CI: Confidence intervals
CT: Computed tomography
cM-stage: Clinical M-stage
cN-stage: Clinical N-stage
cT-stage: Clinical T-stage
DC: Discordant nodal status
dCRT: Definitive chemoradiotherapy
EMR: Endoscopic mucosal resection
EUS: Endoscopic ultrasound
GOJ: Gastro-oesophageal junction
HR: Hazard ratio
IQR: Interquartile range
MDT: Multi-disciplinary team
NACRT: Neoadjuvant chemoradiotherapy
NACT: Neoadjuvant chemotherapy
PET: Positron emission tomography
UK: United Kingdom
