# Supplementary material for: Discordant nodal staging identifies intermediate-risk group for overall survival in patients with cT3 oesophageal adenocarcinoma
Source: Eur Radiol. 2020 Feb 13;30(6):3429–37. doi: 10.1007/s00330-019-06642-6 (PMC7248017; doi:10.1007/s00330-019-06642-6)
Supplement: Supplementary file 1 — (DOCX 24 kb) [file 330_2019_6642_MOESM1_ESM.docx]

**Supplementary Material**

**Discordant Nodal Staging Identifies Intermediate Risk Group for Overall Survival in Patients with cT3 Oesophageal Adenocarcinoma**

**Materials & Methods**

The following PET/CT and EUS protocols were used during routine reporting of the clinical examinations. These details have previously been published in Foley et al [1].

PET/CT protocol

Patients were fasted for at least 6 hours prior to tracer administration. Serum glucose levels were routinely checked and confirmed to be less than 7.0 mmol/L prior to proceeding with imaging. Patients received a dose of 4 MBq of ^18^F-FDG per kilogram of body weight. Uptake time was 90 minutes, which is standard at our institution. ^18^F-FDG PET/CT imaging was performed with a GE 690 PET/CT scanner (GE Healthcare). CT images were acquired in a helical acquisition with a pitch of 0.98 and a tube rotation speed of 0.5 seconds. Tube output was 120 kVp with output modulation between 20 and 200 mA. Matrix size for the CT acquisition was 512 x 512 pixels with a 50cm field of view. No oral or intravenous contrast was administered. PET images were acquired at 3 minutes per field of view. The length of the axial field of view was 15.7 cm. Images were reconstructed with the ordered subset expectation maximisation algorithm, with 24 subsets and 2 iterations. Matrix size was 256 x 256 pixels, using the VUE Point ™ time of flight algorithm.

EUS protocol

All EUS examinations were performed in 3 centres by 4 endosonographers. At the host institution, an initial endoscopic examination was performed using a 9 mm diameter Olympus Paediatric gastroscope (Olympus) to assess the degree of oesophageal luminal stenosis. Patients with an estimated oesophageal luminal diameter <15 mm underwent examination using the smaller-diameter MH-908 oesophagoprobe, and where there was no luminal stenosis, the standard UM-2000 echoendoscope was used (Olympus). The type of echoendoscope used was at the discretion of the endoscopist. The primary oesophageal tumour was assessed, together with an evaluation of peri-oesophageal and peri-gastric structures as described previously. [2] The criteria for malignant lymphadenopathy specified a hypo-echoic pattern, spherical contour, distinct border, and short axis diameter of 6 mm or more.

**References**

1. Foley KG, Christian A, Fielding P, et al (2017) Accuracy of contemporary oesophageal cancer lymph node staging with radiological-pathological correlation. Clin Radiol 72:e691–e697. https://doi.org/10.1016/j.crad.2017.02.022

2. Bowrey DJ, Clark GW, Roberts SA, et al (1999) Endosonographic staging of 100 consecutive patients with esophageal carcinoma: introduction of the 8-mm esophagoprobe. Dis Esophagus 12:258–263
